# Sleep as the Foundation of Brain Health

**DOI:** 10.1055/a-2566-4073

**Published:** 2025-04-25

**Authors:** Abubaker Ibrahim, Birgit Högl, Ambra Stefani

**Affiliations:** 1 Department of Neurology, Medical University Innsbruck, Innsbruck, Austria

**Keywords:** neurodegeneration, stroke, sleep deprivation, circadian rhythm, glymphatic system, memory

## Abstract

Sleep is a vital function, taking about one-third of a human lifetime, and is essential for achieving and maintaining brain health. From homeostatic neurophysiology to emotional and procedural memory processing to clearance of brain waste, sleep and circadian alignment remain paramount. Yet modern lifestyles and clinical practice often dismiss sleep, resulting in profound long-term repercussions. This chapter examines the roles of sleep and circadian rhythms in memory consolidation, synaptic plasticity, and clearance of metabolic waste, highlighting recent advances in neuroscience research. We explore how insufficient and disordered sleep—a public health concern—can impair cognition, escalate neurodegenerative risks, and compromise neurovascular integrity, thereby impacting brain health. These findings underscore the need for comprehensive screening for disturbed sleep and targeted interventions in clinical practice. Emerging interventions and AI-driven technologies may allow early detection and personalized and individualized treatments and improve outcomes. Overall, this chapter reaffirms that healthy sleep is indispensable at any level of neurological disease prevention—on par with the role of diet and exercise in cardiovascular health—and represents the foundation of brain health.

Sleep health and circadian rhythm regulation are fundamental to human health, playing a critical role in optimizing brain function, mental well-being, immune resilience, and cardiometabolic health. Sleep is an active process facilitating several functions essential for brain health, including memory consolidation, 1 synaptic plasticity, 2 and the clearance of neurotoxic waste products through the glymphatic system. 3 Despite its vital role, over half of the world's adult population falls short of the recommended 7 to 9 hours of nightly sleep—with profound consequences for brain health 4 5 6 ( Fig. 1 ).

**Fig. 1:**
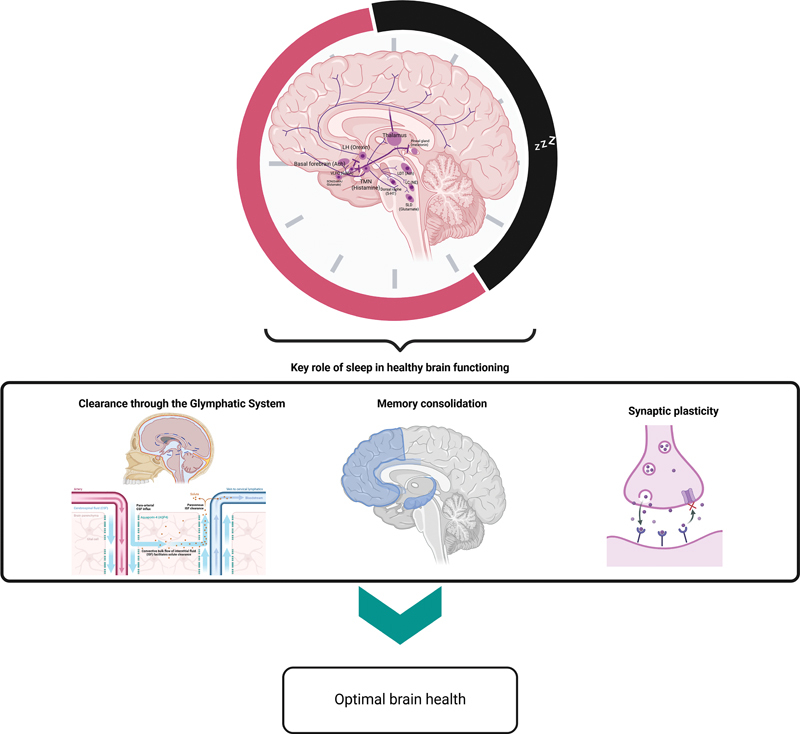
**Healthy sleep.** Schematic overview highlighting the essential role of sleep in maintaining healthy brain function. The circular element at the top illustrates a 24-hour cycle, with the pink segment representing wakefulness and the black segment representing sleep. Inside the circle, a stylized brain indicates key structures involved in sleep regulation (mainly in the hypothalamus and brainstem). Below, three panels depict critical processes that occur, or are enhanced, during sleep: (1) clearance through the glymphatic system, (2) memory consolidation, and (3) synaptic plasticity. Ach, Acetylcholine; LC, Locus Ceruleus; LH, Lateral Hypothalamus; SLD, Sublaterodorsal Nucleus; TMN, Tuberomammillary Nucleus; LDT, Laterodorsal tegmental Nucleus; VLPO, Ventrolateral preoptic nucleus.

Sleep serves as a cornerstone for preventing and better managing various neurological disorders ( Fig. 2 ). Sleep–wake disturbances are closely associated with major neurodegenerative diseases (NDDs), with sleep disorders, alterations in sleep architecture, and circadian rhythm disruptions often preceding their clinical onset. 7 Similarly, sleep disturbances are highly prevalent among post-stroke patients. 8 Notably, even during the developmental stages, sleep loss reduces brain size and produces long-lasting behavioral, morphological, and biochemical abnormalities in later life. 9 Therefore, not only is sleep research paramount in neuroscience, but clinicians also have a unique opportunity to transform patient outcomes and enhance quality of life by making sleep assessment a core component of their practice.

**Fig. 2:**
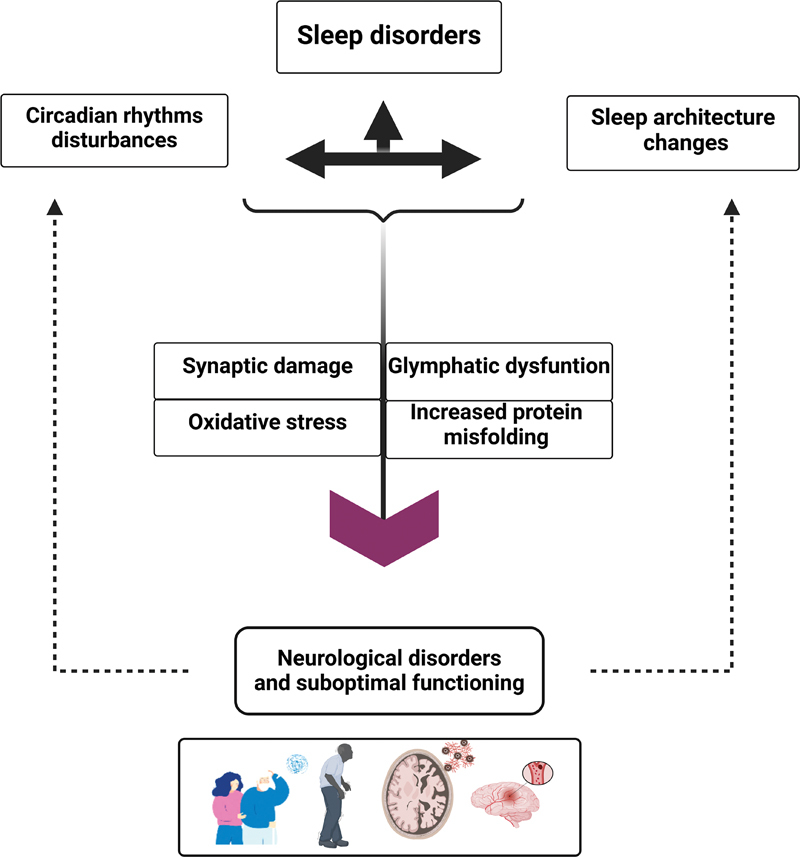
**Disturbed sleep and its consequences on brain health.** Schematic representation of how sleep disorders, circadian rhythm disturbances, and sleep architecture changes can negatively affect brain health. These have a bidirectional relationship and converge to impair several key processes in the brain, including synaptic damage, oxidative stress, glymphatic dysfunction, and increased protein misfolding. The downstream consequence of these disturbances is an elevated risk for neurological disorders (e.g., dementia, Parkinson's disease, and stroke), illustrated at the bottom.

## Sleep Physiology and Brain Health: Mechanisms, Homeostasis, and Implications

### Two-Process Model and Neuronal Circuits Regulating Sleep

The two-process model proposed by Borbély more than four decades ago still offers an important conceptual framework for understanding sleep–wake regulation. 10 The model proposes that sleep regulation is governed by the interaction of a homeostatic process, which builds up during wakefulness and decreases during sleep (Process S), and a circadian process driven by the body's circadian pacemaker, aligning sleep and wakefulness with the 24-hour light–dark cycle (Process C). 11 Brain nuclei involved in sleep–wake regulation are located within the brainstem and the hypothalamus.

The accumulation of endogenous sleep-promoting substances during the day, such as adenosine, activates the ventrolateral preoptic area (VLPO), which is well described in rodents, and the median preoptic nucleus (MnPO). 12 The rodent VLPO and MnPO, which mainly produce gamma-aminobutyric acid (GABA), act on different neurotransmitters and circuits, inhibiting wake-promoting regions ( Fig. 1 ). 12 Other neuronal circuits promoting non-rapid eye movement (NREM) sleep include the parafacial zone in the brainstem (GABA/glycine) and the nNOS neurons in the cortex (nitric oxide), 13 whereas neurons involved in regulating rapid eye movement (REM) sleep are mainly located in the pons. 12 14 Additionally, at night, the absence of light reduces suprachiasmatic nucleus (SCN) activity, lifting the inhibition on melatonin production that the SCN imposes during the day, allowing melatonin synthesis to occur. 15 Melatonin, in turn, enhances the activity of the VLPO and reduces the activity of arousal-promoting brain regions, which promotes sleep.

## Circadian Rhythms and Brain Health

Circadian rhythms, the intrinsic 24-hour cycles that regulate various physiological processes, play a crucial role in maintaining brain health and function. These rhythms are orchestrated by the SCN in the hypothalamus, which serves as the master clock, synchronizing circadian rhythms throughout the body, including those in the brain. The circadian rhythms are self-sustained, meaning that they exist in the absence of any exogenous signals. However, they can be entrained by environmental factors and are influenced by genetics. 16 Light is the primary stimulus (Zeitgeber) for entraining the SCN rhythm period, along with other environmental factors such as physical activity and temperature. Additionally, light suppresses the secretion of melatonin, a hormone that facilitates the transition to sleep, synthesized in the pineal gland with its receptors located in the SCN, as mentioned earlier. 15 The collective work of Jeffrey Hall, Michael Rosbash, and Michael Young demonstrated that individual cells also have a circadian molecular rhythm, which is regulated via a transcriptional–translational feedback loop. Transcription factors such as circadian locomotor output cycles kaput (CLOCK) or brain and muscle ARNT-like protein 1 (BMALI1) regulate the expression of genes such as the encoding period (PER) and the cryptochrome (CRY) that, once translated, inhibit their own transcription. 17 Furthermore, circadian rhythms also experience changes with aging; the timing of sleep onset is relatively earlier in childhood compared with adulthood and shifts later during adolescence, returning to being earlier again in older adults. 17 These changes are also observed at a molecular level. 18 Aging is also associated with a decrease in sensitivity to environmental cues (e.g., reduced response to light due to increased prevalence of ophthalmological conditions 19 ), aligning the circadian rhythms to the natural day/night cycle. Disruptions in circadian rhythms have been linked to neurological disorders, highlighting their significance in brain health, as we discuss in the following sections. Perhaps the most directly affected group is shift workers, as 10% suffer from shift work sleep disorder and are at risk of significant health consequences. 20 Furthermore, a recent meta-analysis showed that shift work was associated with an increased incidence of dementia. 21 Of note, the implications of circadian rhythms disturbances extend beyond cognitive decline; they also encompass metabolic and immune dysregulation, which can further compromise brain health. For example, the interplay between circadian rhythms and the gut microbiome has been identified as a critical factor influencing overall health, including mental well-being. 22

Melatonin, beyond its role in regulating circadian rhythms, has been suggested to have neuroprotective properties, particularly through its interactions with mitochondrial dynamics and cytoprotection. 23 It acts as an antioxidant, scavenging reactive oxygen species (ROS) and enhancing the activity of antioxidant enzymes. Melatonin and its metabolites reduce oxidative damage in particular in the mitochondria, reducing also mitochondria-related apoptosis, contrasting aging-related mitochondrial dysfunction. 24 In line with that, age-related declines in melatonin levels are associated not only with mitochondrial dysfunction but also with increased neuroinflammation, contributing to the pathophysiology of neurodegenerative diseases. 23 This makes melatonin a potential therapeutic candidate for age-related brain disorders.

## Sleep Architecture Across the Lifespan

The structure of sleep is broadly categorized into two primary states: non-rapid eye movement (NREM) sleep and rapid eye movement (REM) sleep. NREM sleep is further subdivided into three distinct stages: N1, N2, and N3, with N3 being characterized as slow-wave sleep. Each stage of sleep exhibits unique electroencephalographic (EEG) patterns, eye movement characteristics, and variations in muscle tone. The progression between NREM and REM sleep occurs in a cyclical manner throughout the sleep period. 25 The time course of process S is characterized by an exponential decline in slow-wave activity during both baseline sleep and recovery sleep following sleep deprivation. REM sleep, on the other hand, is mainly regulated by the circadian pacemaker. 11

Sleep architecture changes across the lifespan; for example, REM sleep is predominant in infants and subtly decreases with age, while the percentage of N3 sleep decreases linearly by approximately 2% per decade of life, plateauing after the age of 60. Consequently, there is an increase in N1 and N2 sleep and wake after sleep onset. 26 Furthermore, sex differences exist, as the decline in N3 sleep is slower in women compared with men. 27 These changes in sleep architecture are accompanied by hormonal changes, as the decline of N3 sleep from early adulthood to midlife is paralleled by a major decline in growth hormone secretion, and increased sleep fragmentation related to aging is associated with higher cortisol levels. 28

### Adenosine: A Key Regulator of Sleep and Cognitive Health

Adenosine plays a crucial role in the regulation of sleep, acting as a homeostatic sleep factor that accumulates during wakefulness (thereby contributing to factor S) and promotes sleep through its interaction with specific adenosine receptors, primarily A _1_ and A _2A_ receptors. 29 Adenosine mediates a glial–neuronal circuit linking glial metabolic state (modulated by adenosine kinase in astrocytes) to neural-expressed sleep homeostasis. 30 The extracellular accumulation of adenosine in the basal forebrain and preoptic areas during prolonged wakefulness enhances sleep pressure, leading to increased sleep propensity. The sleep-promoting effects of adenosine are further evidenced by the observation that its receptor agonists can increase NREM sleep in rats 31 and cats. 32 The stimulation of A _1_ adenosine receptors induces EEG changes that are similar to EEG profiles observed after sleep deprivation. 33 On the other hand, antagonists like caffeine, the most consumed stimulant, counteract these effects by blocking adenosine receptors (non-selectively), thereby increasing alertness and reducing the drive for sleep. 34 The mean half-life of caffeine is about 5 hours, 35 and when humans consume approximately 200 mg of caffeine in the early evening, the endogenous melatonin rhythm is delayed by around 40 minutes. Additionally, one study indicated that caffeine lengthens the circadian rhythms and causes a phase delay ex vivo. 36 Therefore, antagonizing the effect of adenosine could also affect sleep through changes in circadian rhythms (Process C). Furthermore, adenosine plays a role in memory performance. For example, the short-term memory impairment induced by scopolamine could be prevented by A _1_ and A _2A_ antagonists, while the activation of A _2A_ receptors is sufficient to trigger memory impairment in mice. 37 Additionally, one study showed that administration of caffeine enhanced memory consolidation but not retrieval for up to 24 hours according to an inverted U-shaped dose–response curve. 38 Moreover, chronic caffeine consumption was associated with a reduced risk of dementia progression in patients with mild cognitive impairment (MCI), and epidemiological studies found an association between coffee consumption and a lower incidence of Alzheimer's disease (AD). 39 40 These effects are primarily attributed to the antagonism of adenosine receptors. 41 In line with these findings, a recent study showed that blockade of A _2A_ receptors could reverse early spatial memory defects in transgenic mouse AD models by promoting synaptic plasticity. 42 Although the precise mechanism by which adenosine receptor antagonism might prevent dementia progression remains unclear, a recent study has shown that A _2A_ receptors are upregulated in brain samples from patients with various tauopathies (including frontotemporal dementia, corticobasal degeneration, and Pick's disease), potentially accelerating neurodegeneration. 43 This observation suggests that caffeine, through its antagonistic action on these receptors, may help counteract such effects and slow the neurodegenerative processes. However, current evidence on the relationship between caffeine and brain health is inconsistent across various studies due to factors such as gender, age, genetic predisposition, and caffeine dosage. In patients with severe hypertension, drinking more than two cups of coffee a day was associated with an increased risk of cardiovascular mortality. 44 Additionally, coffee consumption in higher dosages (≥300 mg) is associated with an increased risk of neuropsychiatric symptoms in individuals with mental disorders or dementia. 45 46 Daytime caffeine consumption was associated with reduced apathy and aberrant motor behavior but more frequent nighttime awakenings in elderly dementia patients, with caffeine intake after 6 pm significantly contributing to sleep disturbances 47 and reported improved sleep after eliminating caffeine. 48 A study on habitual caffeine consumers found that 10 days of caffeine intake led to reduced gray matter (GM) volume in the medial temporal lobe, independent of sleep depth, suggesting that daily caffeine consumption may induce neural plasticity in this region. 49 Similarly, another study on 36 healthy adults found that 5 days of sleep restriction led to increased gray matter in several brain regions, while caffeine consumption during this period resulted in GM reductions, particularly in the thalamus and prefrontal cortex. The findings suggest that sleep restriction alone may trigger adaptive GM changes, whereas caffeine interferes with this process, potentially through mechanisms independent of A _1_ adenosine receptor availability. 50

In summary, adenosine regulates sleep, circadian rhythms, and cognition, while caffeine, as its antagonist, enhances alertness but can also disrupt sleep and alter brain structure, with still unclear effects on neurodegenerative processes.

## Memory Consolidation and Synaptic Homeostasis During Sleep

Several studies showed that memory performance improves significantly after a whole 8-hour sleep at night 51 52 53 ; moreover, 1- to 2-hour naps 54 55 or even 6-minute naps were shown to provide memory benefits, 56 with longer durations yielding more benefits. 57 Interestingly, for the largest benefits, sleep should happen relatively close to the learning process, as better results on declarative memories have been reported if sleep happens 3 hours compared with 10 hours after learning. 58 59 Not only the duration of sleep but also the different sleep stages play a role in memory consolidation, with different sleep stages affecting different types of memory. NREM sleep is thought to facilitate declarative, hippocampus-dependent memories (e.g., learning facts), whereas REM sleep is thought to facilitate consolidation of memories that are non-declarative (procedural and emotional aspects, e.g., driving a bike). 60 Notably, some studies showed that declarative and non-declarative memory consolidation can occur at any sleep stage. 61 62

Mechanisms involved in memory reinforcement during sleep include memory reactivation and synaptic consolidation. 57 Compelling evidence of the former emerged from a study where participants learned spatial locations paired with an odor. When the odor was reintroduced during N3 but not during REM sleep, it led to a notable enhancement of spatial memory. 63 Moreover, in rats, it was shown that spatial–temporal patterns of neuronal firing occurring during learning are reactivated in the same order during subsequent slow-wave sleep. 64 Slow-wave sleep and sleep spindles are thought to initialize long-term potentiation (LTP) 65 and aid in synaptic consolidation during subsequent REM sleep. Some studies showed a positive correlation between spindle density and post-sleep memory improvement. 66

Moreover, specific neurotransmitters are essential for memory processes during sleep. For instance, acetylcholine has been shown to facilitate memory consolidation during NREM sleep by stabilizing neural network dynamics and enhancing synaptic plasticity. 67 Additionally, the expression of clock genes, such as Per1, has been implicated in regulating memory consolidation during the day, suggesting that the timing of sleep relative to the circadian cycle can influence memory outcomes. 68 Thus, NREM and REM sleep seem to play complementary roles, interacting with circadian cycles, which ultimately optimize memory consolidation.

## Sleep and Waste Clearance

The glymphatic system is a recently discovered waste clearance pathway in the brain, primarily active during sleep. 69 It is a functional system analogous to the lymphatic system but unique to the central nervous system, as the brain lacks conventional lymphatic vessels. This system operates through a network of perivascular spaces that allow cerebrospinal fluid (CSF) to flow through the brain, effectively flushing out waste products that accumulate during wakefulness. The glymphatic system's perivascular tunnels connect directly to the brain's subarachnoid spaces, where cardiac rhythm–driven arterial pulsations propel CFS flow. 70 The organizational units of this system are astrocytes and the water channel Aquaporin 4 (AQP4), which face the vessel wall. The observation that sleep and the glymphatic system are related came initially from animal models, whereby sleep was associated with a 60% increase in the interstitial space, resulting in a significant increase in the convective exchange of CSF with interstitial fluid. 71 Indeed, CSF tracer influx was found to be correlated with EEG slow-wave activity, which means that the glymphatic clearance is most active during N3 sleep (which physiologically corresponds to the first hours of sleep). 72 Moreover, glymphatic flow was shown to be regulated by circadian rhythmicity. 73 In humans, it was found that even a single night's sleep deprivation significantly impairs cerebral clearance. 74 The implications of glymphatic dysfunction are particularly interesting in the context of neurodegenerative diseases. By analyzing multimodal data from the AD Neuroimaging Initiative project, it was shown that coupling between the global functional magnetic resonance image (fMRI) signal and CSF influx is correlated with AD-related pathology. 75 Furthermore, interstitial fluid's convective fluxes increase the amyloid-β clearance rate during sleep. 71 However, one recent study has reported findings that challenge the notion of consistently enhanced glymphatic flow during sleep. This study showed that in mice, brain clearance is markedly reduced, not increased, during sleep and anesthesia. 76 This might, however, be related to methodological aspects, e.g., the dye injection technique used, which can affect brain clearance, impacting the findings and methods applied to measure brain clearance. Therefore, although the concept of the glymphatic system is very promising to improve the understanding of neurodegeneration pathophysiology, no methodology has been established to clearly and directly assess its function, and more research is needed in this area.

## Consequences of Sleep Deprivation on Brain Health

The optimal sleep amount recommended by the American Academy of Sleep Medicine is 7 to 9 hours per night. 5 There is an inverted U-shaped relationship between health outcomes and sleep durations, with sleep duration of less than 6 hours or more than 9 hours being associated with all-cause mortality, older phenotypic age, and increased likelihood of depression. 77 78 79 Sleep deprivation, broadly defined as an insufficient amount of sleep, can manifest in two main forms: acute and chronic. Acute sleep deprivation involves a short-term period—usually 24 to 48 hours—of severely restricted or entirely missed sleep, such as pulling an “all-nighter” to meet a deadline. By contrast, chronic sleep deprivation arises from a longer-term pattern of consistently limited sleep over weeks or months. Both forms can yield profound negative consequences on brain health. Numerous studies showed a decrease in attention and working memory after acute sleep deprivation; these include findings such as slower reaction time, reduced vigilance, and lower performance in serial addition/subtraction tasks. 80 81 Sleep deprivation increases adenosine levels in the basal forebrain, thereby inhibiting the cholinergic system, which plays a role in memory modulation, as mentioned earlier. 82 83 Regarding clearance of brain waste products, one study showed that sleep deprivation increased overnight amyloid-β38, amyloid-β40, and amyloid-β42 levels by 25 to 30% as measured in the CSF. 81 Using positron emission tomography (PET), another study showed that sleep deprivation increases amyloid-β in the hippocampus and thalamus, an increase which was associated with mood worsening; moreover, the study found an inverse association between amyloid-β burden in subcortical areas and reported night sleep hours. 84 Beyond the acute effect of sleep deprivation, a recent study showed that persistent short sleep duration at ages 50, 60, and 70 compared with persistent normal sleep duration was associated with a 30% increased dementia risk. 85 Moreover, reduced sleep efficiency (defined as the percentage of the time in bed spent sleeping) was associated with incident risk of all neurodegenerative diseases. 86 Another recent study showed that sleep regularity seems to be a stronger predictor of mortality than sleep duration. 87 Although more data are needed to disentangle the complex relationship between sleep duration and brain health, these studies highlight the critical role of healthy sleep in maintaining optimal brain health.

## Sleep in Neurodegenerative and Neurovascular Diseases

### Sleep Disorders and Neurodegenerative Diseases

As mentioned earlier, since sleep and circadian rhythms rely on coordinated functions across multiple regions, nuclei, and neurotransmitters in the central nervous system, it follows that neurodegenerative conditions can easily disrupt these interconnected pathways, thereby contributing to sleep disturbances. In AD, for instance, a reduction in the number of neurons within the SCN, 88 combined with diminished synchrony among various circadian oscillators in the brain, 89 is frequently linked to daytime sleepiness, sleep apnea, insomnia, and fragmented sleep patterns. 90 Likewise, very early in the course of Parkinson's disease (PD) the centers responsible for sleep–wake regulation, as well as the expression of clock genes, are impacted, leading to sleep disturbances in nearly all patients. 91 The most common disorders include rapid eye movement sleep behavior disorder (RBD), insomnia, restless legs syndrome (RLS), and sleep-related breathing disorders. Moreover, excessive daytime sleepiness is common in patients with PD. 91 Sleep problems are not confined to these two prevalent neurodegenerative disorders but also affect patients suffering from other neurodegenerative diseases, such as dementia with Lewy bodies, multiple system atrophy, amyotrophic lateral sclerosis, progressive supranuclear palsy, Huntington's disease, and spinocerebellar ataxias. 7

Importantly, sleep disturbances are among the critical early markers indicating the onset of neurodegeneration. The best example is RBD, which is characterized by abnormal behaviors, jerks, and/or vocalizations during REM sleep, and the absence of physiological muscle atonia during REM sleep. Longitudinal studies have reported that the vast majority of patients with isolated RBD (i.e., RBD not secondary to a manifest NDD, other neurological diseaser or other sleep disorders such as narcolepsy) eventually develop an α-synuclein-related neurodegenerative disorder (i.e., PD, dementia with Lewy body and multiple system atrophy), whereby the risk for developing neurodegenerative diseases was 33.5% at 5 years follow-up, 82.4% at 10.5 years, and 96.6% at 14 years. 92 Additionally, in patients with isolated RBD, there is a frequent occurrence of subtle prodromal neurodegenerative abnormalities such as hyposmia, subtle motor deficits, constipation, and orthostatic hypotension, along with abnormalities detected on various neurophysiological and autonomic tests as well as on neuroimaging and biofluids (including the presence of pathological α-synuclein aggregates). 93 94

Another well-investigated example of the link between sleep and neurodegeneration is sleep-disordered breathing (SDB), characterized by recurrent apneas and hypopneas frequently leading to recurrent arousals from sleep, which has been associated with dementia. The earliest longitudinal evidence came from a community study, where women with SDB were more likely to develop MCI (adjusted HR 1.85; 95% confidence interval (CI) 1.11–3.08) after 5 years follow-up. 95 Similarly, in community-dwelling men participating in the Osteoporotic Fractures in Men Study (MrOS), nocturnal hypoxemia was associated with global cognitive decline both cross-sectionally and after 3.4 years. 96 97 In the AD Neuroimaging Initiative cohort, patients with SDB were younger at MCI onset (72–77 versus 82–89 years), or AD dementia onset (83 versus 88 years) after 3 years follow-up, compared with participants without SDB. Moreover, continuous positive airway pressure (CPAP) use was associated with older age at MCI onset. 98 In the Atherosclerosis Risk in Communities Study, initially no association was found between SDB and incident risk of dementia after 15 years. However, a later study from the same cohort, including more participants, indicated that the relationship might not be linear as severe OSA (≥30 apnea–hypopnea events/hour) versus no OSA (<5 apnea–hypopnea events/hour) was associated with a higher risk of all-cause dementia (risk ratio 2.35). 99 A meta-analysis covering 4.3 million individuals indicated that those with SDB were 26% (risk ratio, 1.26; 95% CI, 1.05–1.50) more likely to develop cognitive impairment. 100 In a sample of 53,000 Medicare beneficiaries with SDB, PAP treatment and adherence were associated with lower odds of incident diagnosis of AD (odds ratio [OR] 0.78, 95% CI, 0.69–0.8). 101 Furthermore, imaging studies in cognitively asymptomatic individuals with SDB showed medial temporal lobe atrophy, which may increase the risk of developing memory impairment. 102 Recently, metrics other than the classical apnea–hypopnea index, such as hypoxic burden, were shown to predict cognitive dysfunction in sleep apnea patients. 103

Besides SDB, other sleep disorders and disturbances, such as insomnia, excessive daytime sleepiness, and sleep fragmentation, predate the diagnosis of NDDs. 104 105 106 Furthermore, beyond manifest sleep disturbances, we found that early sleep architecture changes, as measured by polysomnography, are present up to 12 years before the diagnosis of NDDs. These include reduced REM sleep, N3 sleep, sleep efficiency, as well as increased wake in sleep period time. 86 Similarly, in a subset of the Framingham Heart Study (FHS), lower REM sleep percentage and longer REM sleep latency were both associated with a higher risk of incident dementia after 12 years. 107 Additionally, in the MrOS study lower REM sleep percentage and lower α/theta ratio in sleep EEG were associated with incident PD after a median follow-up of 9.8 years. 108

Circadian disturbances are also prevalent in NDDs and are associated with their incidence. For example, these patients may exhibit increased nighttime activity and reduced daytime activity, and in some cases even experience a complete reversal or disruption of their normal 24-hour rest–activity cycle. AD patients also experience increased neuropsychiatric symptoms around sunset, referred to as “sundowning,” which is partly attributed to circadian misalignment. 109 To understand circadian changes in patients with NDD, it is important to distinguish circadian phase from circadian amplitude. The former refers to the timing of specific points within the roughly 24-hour cycle—such as when melatonin levels begin to rise or when core body temperature reaches its lowest point. 110 Circadian amplitude, on the other hand, reflects the intensity of the rhythmic variation—often quantified as the difference between the maximum and minimum levels of a measured circadian parameter (like melatonin concentration or body temperature). 110 PD is characterized by a reduction in the circadian amplitude without shifts in circadian phases. 111 In AD, rest–activity rhythm fragmentation 112 and circadian shifts prevail. Regarding the latter, literature findings are mixed, with one study showing phase delay among AD patients 79 and others finding phase advance. 113 Of note, it is still unclear whether neurodegeneration is a consequence or a cause of circadian disturbances, or both. Using data from the UK Biobank and MrOS, variables reflecting circadian rhythm disruption were derived from the accelerometry data and showed that disturbed circadian rhythm is associated with all-cause dementia, PD, and anxiety disorders 6 to 11 years before their clinical onset. 114 115 Another study found that preclinical AD was associated with rest–activity rhythm fragmentation, and that aging was associated with circadian dysfunction independently of preclinical AD pathology (assessed with PET imaging and amyloid-β pathology). 116 Additionally, a randomized controlled double-blinded study showed that long-term application of bright light treatment (+/− 10,000 lux) reduced cognitive decline by a modest 5% and improved depressive symptoms, while the combination of bright light treatment with melatonin improved sleep efficiency and reduced nocturnal restlessness. 117 In PD patients, bright light exposure (10,000 lux) improved daily activity and reduced daytime sleepiness. 118 Blue light exposure early in the day not only suppresses daytime melatonin but also enhances serotonin levels via the retino-raphe pathway, where photoreceptor signals from the retina stimulate the dorsal raphe nucleus. This process enhances tryptophan metabolism, leading to increased serotonin availability, which is later converted into melatonin in the pineal gland. 119 As a result, natural sunlight exposure during the day ensures optimal serotonin levels, directly influencing nighttime melatonin production. Furthermore, infrared light has been identified as a promising intervention for neurodegenerative diseases, as it stimulates cytochrome c oxidase (complex IV) in the electron transport chain, enhancing ATP production and cellular energy metabolism. 120 This process also facilitates the reduction of oxidative stress and supports mitochondrial resilience, reinforcing its potential therapeutic role in aging and neuroprotection.

Insights from animal models also provide evidence of circadian disruption being linked to NDDs pathology. For instance, a mouse model of amyloidosis-β with deletion of the core clock gene Bmal1 has demonstrated that loss of central circadian rhythms leads to disruption of daily hippocampal interstitial fluid amyloid-β oscillations and accelerates plaque accumulation. 121 Another study found that orexin regulates the hippocampal clock and circadian oscillation of AD risk genes. 122 Overall, circadian science is evolving and provides new insights into early detection and modulation of NDDs.

### Sleep and Stroke

Cardiovascular diseases remain the leading cause of all-cause mortality. 123 In the context of sleep and neurovascular health, the most relevant sleep disorder is SDB, as it affects around 20% of the general population. SDB is related to wake-up stroke through mechanisms including non-dipping blood pressure and impaired cerebral hemodynamics. 124 On the other hand, SDB is common in stroke patients; a meta-analysis of 2,343 ischemic or hemorrhagic stroke and TIA patients found the frequency of SDB with AHI > 5 to be 72% and with AHI > 20 to be 38%. 125 In both the Wisconsin Sleep Cohort Study (1,189 subjects followed up for 4 years) and the Sleep Heart Health Study (5,422 participants followed up for 8.7 years), there was a 2- to 3-fold increase in the risk of stroke for subjects with untreated SDB. 126 127 Silent brain infarctions on MRI were more common among patients with moderate to severe sleep apnea than among control subjects or patients with mild sleep apnea. 128 Importantly, this is an easily modifiable risk factor, as CPAP therapy with good adherence, which is the gold standard for SDB, is associated with stroke risk reduction. 129 Notably, poor compliance with PAP therapy negates benefits concerning stroke risk reduction. Beyond SDB, other sleep disorders, such as RLS, seem to possibly occur post-stroke in the setting of lesions in the basal ganglia/corona radiata. 130 RLS is also likely linked to a higher prevalence of coronary artery disease and cardiovascular disease, and this relationship appears to be stronger in individuals who experience more frequent or severe RLS symptoms. 131 Furthermore, a recent study showed an association between insomnia and objective (<6 hours confirmed by polysomnography) but not subjective short-sleep duration and incident cardiovascular diseases after a median follow-up of 11.4 years. 132 Moreover, persistent insomnia symptoms were associated with an increased risk of stroke in younger adults. 133

Sleep changes are also frequent post-stroke, with poor sleep quality affecting up to 53% of stroke patients. 134 Sleep architecture was shown to be significantly affected in 104 post-stroke patients compared with 162 controls; changes included reduced total sleep time and sleep efficiency, and increased wakefulness. Additionally, the percentage of REM sleep was shown to be negatively associated with stroke severity. Of note, in the same study, sleep quality and sleep architecture improved but did not normalize during the 3 months after stroke. 135

In 437 stroke patients over 2 years follow-up (after one to seven days, one month, three months, twelve months and twenty-four months), the frequencies of excessive daytime sleepiness (EDS), fatigue, and insomnia varied between 10 and 14% for EDS, 22 and 28% for fatigue, and 20 and 28% for insomnia over the five follow-up visits. 136 Additionally, depending on the infarct area, not only RLS but also other sleep disorders such as RBD or hypersomnolence disease can occur, albeit more rarely as small strategic areas need to be affected by the stroke to cause these symptoms. 8 In conclusion, the complex bidirectional relationship between stroke and sleep requires further investigation, given the high prevalence of these diseases and the potential implications of treating sleep disorders in stroke prevention and management.

## Therapeutic Implications and Future Directions

The most straightforward and broadly applicable intervention to safeguard against the deleterious effects of sleep disturbances is maintaining adequate sleep duration and rhythmicity and practicing sound sleep hygiene. Fundamental measures include establishing a consistent bedtime and rise time, avoiding stimulants like caffeine in the late afternoon or evening, and ensuring an environment conducive to rest—cool, dark, and free from excessive noise. Such non-pharmacological strategies can substantially improve sleep quality, reduce nocturnal awakenings, and bolster cognitive function, especially in individuals experiencing early, mild sleep problems. 5 137 Moreover, by raising public awareness of sleep disorders, early detection and intervention become more feasible, reducing the risk of serious complications. Strengthening sleep education and implementing targeted healthcare strategies are, therefore, critical steps toward improving overall sleep health and preventing long-term adverse outcomes impacting brain health. 6

When these general interventions fail to sufficiently address persistent sleep issues, targeted treatments become necessary. For example, those suffering from insomnia can often benefit from Cognitive Behavioral Therapy for Insomnia (CBT-I). This evidence-based and currently first-line approach combines behavioral techniques (e.g., stimulus control, sleep restriction) with cognitive strategies aimed at alleviating anxiety or maladaptive thoughts about sleep. 138 Several studies have shown that CBT-I not only improves sleep continuity and reduces insomnia severity but may also have a protective effect against cognitive decline and reduce the rate of amyloid-β deposition in older adults. 139 Additional interventions, such as melatonin supplementation (to recalibrate the sleep–wake cycle), emerge as promising candidates for mitigating AD pathology. 140 A meta-analysis of 22 randomized controlled trials in patients with AD who received more than 12 weeks of melatonin treatment showed improvements in Mini-Mental State Examination scores. 141 Additionally, higher melatonin levels in community-dwelling adults correlated with lower cognitive impairment. 142 The orexin (hypocretin) signaling pathway offers a compelling frontier for novel therapeutic strategies aimed at optimizing sleep architecture. Orexins regulate wakefulness and stabilize sleep–wake transitions; thus, selective antagonists (orexin receptor blockers) have been effective and are currently available for treating insomnia, 143 with emerging research suggesting, beyond insomnia treatment, potential roles in modulating disease course in AD. 144 PD patients might instead benefit from orexin agonists, 145 as PD is associated with lower orexin levels. 146 As discussed above, treatment of obstructive sleep apnea reduces the risk of neurovascular and neurodegenerative disorders. Newer interventions aiming at directly improving the sleep structure, such as acoustic stimulation (to enhance slow-wave sleep), are promising as they could improve glymphatic clearance and memory consolidation, but research on this topic is still ongoing, 136 137 and longitudinal data to demonstrate such benefits are needed.

## Role of Wearable/Nearable Devices and Artificial Intelligence

In parallel with pharmacologic advances, the steadily increasing availability of wearable and nearable devices and AI-driven technologies for early identification and monitoring of sleep alterations are starting to reshape both clinical practice and large-scale screening. Automated analyses of polysomnography, actigraphy, other devices used in the research field, or even consumer sleep-tracker data can detect subtle, prodromal sleep pattern disruptions—such as sleep fragmentation or microarousals—years before overt neurological symptoms appear. 147 148 149 Additionally, these technologies not only provide continuous measures of multiple parameters, but they also allow applying a new way to score traditional sleep, introducing the concept of “hypodensity” to score sleep in a probabilistic way instead of relying on the traditional sleep stages. 150 Moreover, these technologies are widely available now and are getting more diverse (wearables, nearables, and airables), offering multifaceted and continuous observation for different groups (e.g., those who do not tolerate sleeping with a smart-watch could use a smart-mattress or a smartphone which can detect movement or snoring) with the potential to unlock new knowledge in preventing, diagnosing early, and managing neurological diseases. 151 Furthermore, the emergence of commercial Large Language Models (LLM; e.g., GPT [OpenAI], LLAMA [Meta], Gemini [Google]) offers new ways in which patients can interact with the technology and get direct answers to their complaints, 152 but the thorough validation of these tools is still needed. By integrating these AI insights into personalized and individualized sleep management plans, clinicians may intervene earlier, tailoring therapies that could meaningfully delay disease onset and progression.

## Take-home Messages and Conclusion

Sleep is a cornerstone of brain health and should be prioritized accordingly. Subtle sleep disturbances may precede neurological disease by years, providing a critical window for early intervention. Prompt management of obstructive sleep apnea, insomnia, sleep deprivation, or circadian disruptions reduces the risk of occurrence and disability after the onset of stroke and neurodegenerative diseases. Meanwhile, AI-driven sleep trackers and automated analyses will empower clinicians to detect prodromal and preclinical changes, personalize treatments, and optimize outcomes, helping at the same time raising awareness of the relevance of sleep health in the general population. Maintaining a healthy sleep, through consistency in sleep–wake schedules and adequate sleep duration, underpins brain health and overall well-being.
